# Eastern Canadian Gastrointestinal Cancer Consensus Conference 2023

**DOI:** 10.3390/curroncol30090593

**Published:** 2023-09-04

**Authors:** Essa Al-Mansor, Meghan Mahoney, Maxime Chenard-Poirier, Ravi Ramjeesingh, Vimoj Nair, Erin Kennedy, Gordon Locke, Stephen Welch, Scott Berry, Felix Couture, Elena Elimova, Aaron Pollett, Aamer Mahmud, Brooke Wilson, Dawn Armstrong, Conrad Falkson, Timothy Asmis, Michael Vickers, Rachel Goodwin

**Affiliations:** 1 The Ottawa Hospital Cancer Centre, Ottawa, ON K1H 8L6, Canadargoodwin@toh.ca (R.G.); 2 Faculty of Medicine, Memorial University of Newfoundland, Dr. H. Bliss Murphy Cancer Centre, St. John’s, NL A1B 3V6, Canadac; 3 CHU de Québec—Université Laval, Québec, QC G1R 2J6, Canadafelix.couture@crchudequebec.ulaval.ca (F.C.); 4 Queen Elizabeth II Health Sciences Centre, Halifax, NS B3H 2Y9, Canada; 5 Mount Sinai Hospital, Toronto, ON M5G 1X5, Canada; erin.kennedy@sinaihealthsystem.ca (E.K.);; 6 London Regional Cancer Program, London, ON N6A 5W9, Canada; stephen.welch@lhsc.on.ca; 7 Department of Oncology, Cancer Centre of Southeastern Ontario, Queen’s University, Kingston, ON K7L 5P9, Canada; scott.berry@kingstonhsc.ca (S.B.);; 8 Princess Margaret Cancer Centre, Toronto, ON M5G 2C4, Canada

**Keywords:** guidelines, hepatocellular carcinoma, pancreatic cancer, colon cancer, gastro-esophageal cancer, rectal cancer, chemotherapy, radiation therapy, immunotherapy, surgery

## Abstract

The annual Eastern Canadian Gastrointestinal Cancer Consensus Conference 2023 was held in Quebec City, Quebec 2–4 February 2023. The purpose of the conference was to develop consensus statements on emerging and evolving treatment paradigms. Participants included Canadian medical oncologists, radiation oncologists, pathologists and surgical oncologists from across Ontario, Quebec, and the Atlantic provinces. Consensus statements were developed following rapid review presentations and discussion of available literature. The recommendations proposed here represent the consensus opinions of physicians involved in the care of patients with gastrointestinal malignancies who participated in this meeting.

## 1. Introduction 

The annual Eastern Canadian Gastrointestinal Cancer Consensus Conference 2023 was held in Quebec City, Quebec, 2–4 February 2023. The purpose of the conference was to develop consensus statements on emerging and evolving treatment paradigms. Participants were Canadian medical oncologists, radiation oncologists, and surgical oncologists from across Ontario, Quebec, and the Atlantic provinces. Consensus statements were developed following rapid review presentations and discussion of available literature. The recommendations proposed here represent the consensus opinions of physicians involved in the care of patients with gastrointestinal malignancies who participated in this meeting. 

### Basis of Recommendations 

The existing scientific evidence was presented and discussed at the meeting. Recommendations were formulated within the group and categorized by level of evidence as follows:

- Level I: evidence from randomized controlled trials
- Level II-1: evidence from controlled trials without randomization
- Level II-2: evidence from analytic cohorts or case-control studies, preferably from more than one center or research group
- Level II-3: evidence from comparisons between times or places with and without the intervention
- Level III: Opinion of respected authorities based on clinical experience; descriptive

## 2. Immunotherapy in Advanced Colorectal Cancer

Question 1:

Is there an optimal predictive biomarker for immunotherapy in advanced colorectal cancer (aCRC)?

- Mismatch repair deficiency (dMMR) should be assessed by immunohistochemistry or microsatellite instability (MSI) by DNA sequencing. With the level of evidence to date, either are acceptable tests to predict response to immunotherapy. (Level II-2)
- Assessment of MMR/MSI is required in the first line testing of aCRC. All patients should have timely access to testing of MMR deficiency. (Level III)

Evidence Summary:

Mismatch repair deficiency (dMMR) describes mutations in one or more DNA mismatch repair proteins (MLH1, MSH2, MSH6, PMS2) and is associated with 15% of colorectal cancers (4–5% in the metastatic setting). dMMR may be inherited in an autosomal dominant pattern (i.e., Lynch syndrome) or occur through promoter hypermethylation of MLH1 (sporadic CRC), however inactivation of both alleles is needed for phenotypic expression. These tumors are known as microsatellite unstable or (MSI-high) which refers to expansion or contraction of microsatellite regions leading to increased immunogenicity through dysregulation of key genes [1]. In an early, phase I trial on anti-PD1 antibodies in solid tumors, although overall response rate was low, there was found to be one prolonged complete response in a patient with colorectal cancer who was noted to be MSI-H [2]. This led to further investigations into what is now known to be a key prognostic biomarker in advanced colorectal cancer, as it has been well established that dMMR/MSI-H tumors are responsive to treatment with immune checkpoint blockade. As a result, anti-PD1 antibodies are now the global standard of care to treat colorectal cancers which are dMMR/MSI-H. Given this, the treatment algorithm of advanced colorectal cancer now depends upon dMMR/MSI-H status, and it is recommended that all patients have timely access to this testing by immunohistochemistry or microsatellite instability by DNA sequencing. This testing should be completed on the most recent pathology or prior to the earliest line of treatment with results ideally available at the time of initial systemic therapy consultation.

Question 2:

What is the role of immunotherapy in aCRC?

- Pembrolizumab should be offered to patients with MSI-H, aCRC as the standard first line therapy based on improvements in response rate (RR), progression free survival (PFS) and quality of life (QoL) compared to physician’s choice chemotherapy. (Level I)
- Beyond the first line setting, data from single arm trials show high response rates in patients with dMMR/MSI-H aCRC not previously treated with immunotherapy (IO). (Level II-1)
- There is a lack of data regarding the best treatment for patients with dMMR/MSI-H aCRC that progress on first line single agent IO. Options include clinical trials, chemotherapy, or Encorafenib with Cetuximab (if BRAF/V600E mutation present). (Level III)
- There is currently no role for immunotherapy in microsatellite stable (MSS) advanced colorectal cancer outside the context of a clinical trial. (Level I)

Evidence Summary:

All patients with advanced dMMR/MSI-H colorectal cancer should be exposed to IO at some point in their care, preferably the earliest line of treatment possible, so long as they do not have any contraindications to immunotherapy. KEYNOTE-177 randomized patients with dMMR/MSI-H aCRC to single agent immunotherapy in the first line setting with pembrolizumab versus standard first line chemotherapy (FOLFOX or FOLFIRI +/− bevacizumab or cetuximab). Overall response rates were higher in the pembrolizumab group (43.8% vs. 33.1%) and progression free survival (PFS) was superior to chemotherapy (16.5 vs. 8.2 months respectively, HR 0.60; 95% [CI], 0.45 to 0.80; P = 0.0002). Immunotherapy was well tolerated with fewer significant (grade three or higher) adverse events compared to the chemotherapy arm (22% versus 66%). Median overall survival was not reached in the IO arm (36.7 months in chemotherapy arm, pre-specifiedα of 0.025 not met) and there was a 59% crossover to anti-PD-1/anti-PD-L1 therapy in the chemotherapy group [3]. A phase II study evaluating first-line combination immunotherapy with Nivolumab and Ipilimumab for dMMR/MSI-H advanced CRC reported a two-year overall survival rate of 79.4%. While these results are promising, combination immunotherapy has not been shown to be superior to monotherapy and is associated with higher rates of toxicities as grade 3-4 treatment related adverse events occurred in 22% of patients [4]. In select patients with a high burden of disease, it may be suitable to consider starting with chemotherapy. There are multiple trials ongoing to evaluate the combination of immunotherapy with cytotoxic chemotherapy in the first line setting to further delineate optimal first line therapy. 

Beyond the first line, patients with dMMR/MSI-H aCRC who have not been previously treated with immunotherapy should receive immunotherapy upon progression. In a 2015 study conducted on patients with metastatic, treatment refractory progressive cancer in multiple disease sites, those with dMMR demonstrated an overall response rate of 40% (compared to 0% in those who were MMR proficient) and median overall survival (OS) was not reached. This data provided the initial support for immunotherapy beyond the first line setting and Pembrolizumab was approved by the FDA in 2017 for this indication. Further support was gained following KEYNOTE-164 which was a phase II trial assessing the efficacy of pembrolizumab in treatment refractory, advanced colorectal cancer amongst patients who had received one or two prior lines of treatment and were stratified accordingly. Overall response rates were 33% in both groups. Those pretreated with more than two lines of therapy demonstrated median overall survival of 31.3 months and for patients who had received only one prior line of therapy median overall survival was not reached. Grade three or higher adverse effects were reported in about 15% of patients overall demonstrating a reasonable safety profile [5]. 

It remains unclear what the most appropriate treatment option is for dMMR/MSI-H colorectal patients who progress on immunotherapy. Options should consider BRAF/V600E mutational status (Encorafenib +Cetuximab), chemotherapy or enrollment in a clinical trial. 

Question 3:

What are the important considerations for the management of IO toxicities of GI cancer patients?

- Immunotherapy-mediated adverse events (IMAEs) can occur at any time during the treatment of a patient and can occur even after treatment discontinuation.
- A strong understanding of baseline symptoms is important to distinguish immune-mediated AEs versus normal variation due to the disease.
- If a provincial immune-mediated toxicity management guideline exists in your province, this should be followed.
- Current grading and tracking of IMAEs rely on the use of the National Cancer Institute’s Common Terminology Criteria for Adverse Events (NCI CTCAE V.5.0), in which adverse events are rated from grade 1 to 5, corresponding to mild, moderate, severe, life-threatening and death, respectively.
- In general, grade two or higher toxicity is generally correlated with the need for medical intervention
- Because of the prolonged kinetics of IO, patients that have presented to an emergency room should have timely oncology follow-up after discharge as toxicities may continue to evolve over days to weeks.

Evidence Summary:

In recent years, immunotherapy has been incorporated as a treatment option for many gastrointestinal malignancies, underscoring the importance of optimal management of immune-mediated adverse effects (IMAEs). Given the nature of disease of GI malignancies, it is imperative to establish a strong understanding of each patient’s baseline general symptoms, bowel habits, comorbidities, and functional status such that immune-mediated toxicities can be delineated from disease burden or progression. Understanding the expected chemotherapy side effects from a chemoimmunotherapy regimen is integral to differentiating IMAEs from chemotherapy related side effects. Therefore, assessment and treatment of IMAE should be tailored specifically to patients’ ongoing treatment regimens. The characterization of IMAEs should follow the National Cancer Institute’s terminology criteria for adverse effects, grading toxicities on a scale from 1-5 (grade 1 being mild and 5 life-threatening) [6]. It is recommended that each province utilize guidelines as specified by their region or refer to those established in other reputable Canadian centers (i.e., BC Cancer, Cancer Care Ontario) [7,8]. As grade two toxicities or higher generally require intervention, early consultation with the appropriate medical subspecialty is strongly recommended. Clinicians should be proficient in managing steroid tapers, holding and restarting immunotherapies and monitoring for immune mediated toxicities that may evolve over time. In any case, close follow-up is required even beyond the date of treatment completion as late toxicities may also occur. 

## 3. Rectal Cancer

Question 1:

What are the general principles in the management of Localized rectal cancer?

- The management of all patients with Localized rectal cancer should be discussed in a multidisciplinary round including colorectal surgeons, radiologists, pathologists, medical oncologists, radiation oncologists & gastroenterologists. (Level III)
- Important components of an informed patient discussion include:
  - Patient’s health status including comorbidities and performance status.
  - Patient values, goals & preferences.
  - Likelihood of requiring a permanent colostomy or temporary ileostomy.
  - Chances of avoidance of TME surgery with non-operative treatment approaches.
  - The frequency and types of assessments required for non-operative management (NOM).
  - The risks of local and distant recurrence with NOM.

Evidence Summary:

Recently, the management localized rectal cancer has evolved by to include strategies aimed at reducing local/distant recurrence and improving PFS & OS. In addition, there have also been efforts investigating non-operative management of rectal cancer. Choosing between these treatment strategies is challenging & depends on the presence of high-risk features, patient’s comorbidities & their preferences to avoid surgery and/or permanent colostomy. Therefore, patient-centered treatment plans should be decided after a thorough multidisciplinary board review by experts in medical oncology, radiation oncology, colorectal surgery, pathology, radiology & gastroenterology [9,10].

Question 2:

What population of patients benefits the most from total neoadjuvant treatment (TNT) and what is the preferred regimen?

- The standard of care for stage II & III rectal cancer patients remains pre-op (chemo)radiation followed by surgical resection. (Level I)
- TNT could be considered for fit selected patients with stage II & III rectal cancer. (Level III)
- TNT strategies are evolving.
- In patients for whom a TNT strategy is being considered and depending on the chosen regimen, the informed discussion should include the potential for an increased risk of locoregional failure, balanced against a disease specific survival improvement and uncertainty regarding long term outcomes.
- Radiotherapy + consolidative chemotherapy (TNT) followed by Surgery is non-inferior to nCRT followed by surgery. (Level I)
- The PRODIGE 23 protocol of neoadjuvant chemotherapy plus preoperative chemoradiation and total mesorectal excision may be considered as a treatment option for selected patients. (Level I)

Evidence Summary:

For locally advanced rectal cancer (LARC), preoperative short-course radiotherapy (SCRT) or long-course radiotherapy administered in combination with fluoropyrimidine-based chemotherapy (CRT), total mesorectal excision (TME), and postoperative adjuvant chemotherapy (ACT) had traditionally been considered standard of care options [11].

For the majority of stage 2-3 rectal cancers, consideration of a TNT approach is recommended in fit, selected patients due to the significant risk of disease recurrence [10]. Patients who are selected for treatment with the Rapido protocol (SCRT + oxaliplatin-based neoadjuvant chemotherapy followed by TME) should be aware of the potential higher risk of locoregional treatment failure (12%) vs. (8%) in conventional therapy. Conversely, the Rapido trial also showed a reduced risk of disease-related treatment failure (23.7%) compared with conventional therapy (30.4%) as well as increase the rate of pathologic complete response with total neoadjuvant therapy (28%) vs. (14%) in conventional therapy [12].

Despite these supporting results from the Rapido trial for a TNT approach, the STELLAR trial that (similar design to Rapido) trial failed to demonstrate this advantage for-of TNT compared to nCRT [13]. Based on the PRODIGE 23 trial, neoadjuvant mFOLFIRINOX + CRT is safe, and showed a higher chance of ypCR rate, as well as improved disease-free survival (DFS), and metastases free survival compared with nCRT followed by TME and ACT, thus it should be taken in consideration in certain patient [14]. 

Question 3:

Is there a role for NOM of rectal cancer to avoid a TME surgery?

- Non-operative management of rectal cancer should be performed at centers with multi-disciplinary cancer management expertise. (Level III)
- Points of discussion for patients who are considering NOM include future bowel function, the quality of clinical trial data, patient preference for avoiding an ostomy, disease free survival, overall survival, toxicity of treatment, chance of TME free survival, intensity & modality of surveillance follow up. (Level III)
- In the largest NOM trial to date, the outcomes of stage II/III low rectal cancer patients showed no difference in DFS compared to historical controls and there was improved clinical complete response (cCR) and TME free survival in favor of nCRT followed by the chemotherapy compared with the reverse. (Level I)
- Based on this data, in patients where NOM is the upfront goal, we endorse CRT followed by consolidative doublet (fluoropyrimidine + oxaliplatin) chemotherapy as the preferred NOM strategy.

Evidence Summary:

NOM is an emerging standard of care for patients with rectal cancer who are interested in organ preservation.

Studies suggest different approaches for NOM/watchful waiting, including interval range of surveillance images as well as other investigations that need to be performed at high-volume facilities devoted to the evaluation and follow-up of these patients [15,16,17].

The OPRA protocol (long course nCRT followed by consolidative doublet (fluoropyrimidine + oxaliplatin chemotherapy) improved the cCR and 3-year TME free survival (53%) compared to induction chemotherapy followed by CRT(41%) [18]. 

After neo-adjuvant therapy has been completed and a thorough multidisciplinary discussion has taken place, patients with a cCR may enter a Wait & Watch (W&W) protocol with the understanding that strict adherence to an intensive surveillance protocol is required. In 2021 international consensus recommendations on key outcomes measures for organ preservation following neoadjuvant chemoradiotherapy in patients with rectal cancer were developed. We endorse these recommendations which include digital rectal examination, endoscopy and MRI every 3-4 months for the first 2 years, then every 6 months for 3 years. They also suggest measuring serum carcinoembryonic antigen levels every 3 months for the first 3 years and then every 6 months for the following 2 years. A surveillance CT of the chest and or abdomen is recommended every 6 months for the first year and then annually for the following 4 years [19]. Patients with mid-distal rectal adenocarcinomas are frequently appropriate candidates for W&W, as the only other treatment options are an abdominoperineal resection or a low stapled/handsewn colorectal/coloanal anastomosis, both of which may have a negative impact on QoL due to potential low anterior resection syndrome and permanent or temporary stoma [20].

## 4. Treatment of End Stage Colorectal Cancer

Question 1:

- What are the emerging options for systemic therapy in the third line setting for metastatic CRC?
- For patients with mCRC who have had 2 previous lines of therapy, adding bevacizumab to trifluridine/tipiracil produces a statistically significant and clinically meaningful benefit in median OS and delay in time to deterioration in Global Health Status. (Level I). This combination can be considered for patients with RAS mutated tumors. (Level III)
- For Ras WT patients who have had 2 previous lines of treatment and have not received EGFR Mab, EGFR Mab should be offered in the third line setting. (Level I)

Evidence Summary:

The current treatment options in the setting of refractory metastatic colon cancer (i.e., those previously treated with 1 or 2 lines of chemotherapy doublet +/− EGFR or VEGF targeted agents) are with multi-kinase inhibitor regorafenib or nucleoside cytotoxic agent trifluridine/tipiracil. Randomized control trials evaluating regorafenib (CORRECT 2013 & CONCUR 2015) demonstrated statistically significant but clinically modest improvements in median overall survival when compared to placebo + best supportive care (6.4 months vs. 5.0, CORRECT & 8.8 months vs. 6.3, CONCUR). Notable adverse effects include hand-foot skin reaction, fatigue, diarrhea, hypertension, and rash [21,22]. Trials evaluating trifluridine/tipiracil (RECOURSE 2015 & TERRA 2018) similarly noted statistically significant but modest clinical improvements in overall survival compared to best supportive care + placebo (mOS 7.1 vs. 5.3 months, RECOURSE & 7.8 vs. 7.1 months, TERRA) [23,24]. When T/T and regorafenib were compared in a 2017 systematic review and network meta-analysis, no significant difference was found in PFS or OS, however toxicity profiles favored trifluridine/tipiracil [25].

In the phase III SUNLIGHT trial, patients with metastatic colorectal cancer who had been previously treated with 2 lines of systemic therapy and had known RAS mutation status were randomized to receive trifluridine/tipiracil alone or with the addition of bevacizumab. 76% of patients had been treated with anti VEGF agents in prior lines of therapy. Patients who received bevacizumab in addition to trifluridine/tipiracil were found to have a significant median overall survival benefit of 10.8 months versus 7.5 months compared with trifluridine/tipiracil alone (HR 0.61, 95% CI [0.49, 0.77], *p* < 0.001). There was also a significant improvement in time to deterioration of global health status in the intervention arm (8.5 vs. 4.7 months, HR 0.5, *p* < 0.001) Hypertension, nausea and neutropenia were the most common adverse effects in the bevacizumab + trifluridine/tipiracil arm. Subset analysis of patients based on prior bevacizumab exposure demonstrated an OS HR of 0.40 for patients with no prior bev exposure and HR 0.72 for patients who had been treated with bev [26].

A global phase III multiregional clinical trial titled FRESCO-2 explored the efficacy of oral tyrosine kinase inhibitor fruquintinib in heavily pretreated patients with metastatic colorectal cancer where the only remaining treatment option was best supportive care compared to placebo. Fruquitinib demonstrates a statistically significant, modest survival benefit compared to best supportive care and placebo (OS 7.4m vs. 4.8m; HR = 0.66, and PFS of 3.7m vs. 1.8m; HR = 0.32). The fruquintinib group had more hypertension, asthenia, and hand-foot syndrome. 

The potential benefits of fruquitinib need to be considered in the context of toxicities and no convincing evidence of delay in deterioration of quality of life [27].

## 5. The Role of Radiation in the Treatment of Pancreatic Cancer

Question 1:

- What is the role of neoadjuvant chemoradiation (nCRT) in borderline resectable pancreatic ductal adenocarcinoma?
- The role of nCRT is controversial and unclear given the lack of disease-free survival or overall survival benefit in the studies completed to date. (Level III)

Evidence Summary:

The evidence in support of neoadjuvant chemoradiation in the treatment of pancreatic ductal adenocarcinoma remains unclear. A Dutch metanalysis found that nCRT following neoadjuvant chemotherapy with FOLFIRINOX did improve R0 resection rates (88.0% vs. 97.6%, *p* = 0.045) however, a difference in median overall survival was not demonstrated (21.6 months for chemotherapy alone and 22.4 months for nCRT) [28]. While PREOPANC-1 demonstrated an improvement in median overall survival benefit for patients who received nCRT compared to up front surgery, (15.7 months vs. 14.3 months), gemcitabine was the concurrent chemotherapy of choice in this trial, which is not the current standard of care and overall survival rates were noticeably shorter than those demonstrated in other trials. Of note, it did suggest that the benefit of nCRT in combination with chemotherapy was more pronounced for patients with borderline resectable disease (HR = 0.67) compared with resectable disease (HR = 0.79) [29]. It is recommended that if planning for nCRT one should consider the treatment toxicities of this therapy in the context of a potential upcoming surgery. Concurrent chemoradiation doses range from 45–50.4 Gy in 25–28 fractions with capecitabine, or 36 Gy in 15 fractions (PREOPANC1). The use of SBRT is not recommended outside of a clinical trial setting, as per Alliance A021501 which did not yield improvements in overall survival when hypofractionated RT or SBRT were used with FOLFIRINOX in the neoadjuvant setting, compared to historical data [30]. Following neoadjuvant chemotherapy, if the patient’s tumor is considered borderline resectable and subject to multidisciplinary discussion, neoadjuvant chemoradiotherapy can be considered to improve the chances of R0 resection. 

Question 2:

What is the role of adjuvant chemoradiation in resected pancreatic ductal adenocarcinoma?

- There is no role for routine chemoradiotherapy in resected pancreatic cancer. (Level I)
- In patients receiving multiagent chemotherapy, the role for routine chemoradiotherapy with high-risk features such as R1 resection is unclear. (Level III)

Evidence Summary:

Although there is no indication for routine adjuvant chemoradiotherapy for pancreatic adenocarcinoma, in the case of incomplete resection (R1), chemoradiotherapy can be proposed after three to six months of adjuvant chemotherapy based on the results of a 2005 metanalysis. Prognostic subgroup analysis in this paper demonstrated a significant difference in the effect of chemoradiation amongst those with positive margins (c^2^ = 4.2, *P* = 0.04), but otherwise there were no significant differences in those receiving chemoradiation over chemotherapy alone [31]. As per ASTRO consensus guidelines, patients with resected pancreatic cancer selected for adjuvant conventionally fractionated RT and chemotherapy should receive 4500–5400 cGy in 180–200 cGy fractions [32]. After receiving multiagent chemotherapy, & if there are high risk features such as R1 resection, multidisciplinary tumor board discussion is warranted to review the role of adjuvant radiation.

Question 3:

What is the role of RT in the palliation of locally advanced or metastatic pancreatic cancer?

- Routine use of chemoradiation in unresectable locally advanced pancreatic cancer is unclear in combination with mFOLFIRINOX or Gemcitabine based chemotherapy. (Level I)
- Chemoradiotherapy can be considered after at least 3 months of chemotherapy in the absence of distant progression for locally advanced disease. (Level III)
- Palliative radiotherapy should be considered for symptom palliation. (Level II)

Evidence Summary:

There is evidence to support the use of chemoradiation (CRT) for patients who do not have progressive cancer after four months of chemotherapy in the setting of locally advanced, pancreatic ductal adenocarcinoma. Hugeut et al, (ASCO 2014) demonstrated that while there is no overall survival benefit, patients who received chemoradiation had less local progression than those in the chemotherapy alone arm (34% vs. 65%, *p* < 0.0001). It was also noted that the CRT group went on to have a longer median time without requiring the reintroduction of chemotherapy (159 vs. 96 days, *p* = 0.05), which may suggest improvements in quality of life [33].

Chemotherapy followed by CRT (50.4 Gy in 28 fractions with capecitabine/gemcitabine) is a recommended regimen. If CRT is planned for locally advanced disease, definitive conventionally fractionated or dose-escalated RT with chemotherapy, 5040–5600 cGy in 175–220 cGy fractions with concurrent chemotherapy is recommended. Dose escalation (boost to 54 Gy) can be considered but the benefit must be weighed against the risk of toxicity.

For selected patients with metastatic pancreatic cancer, palliative RT to either the primary or select metastatic sites for symptom management may be considered. For symptoms such as pain and obstruction, palliative radiotherapy appears to be well tolerated without severe toxicities [34]. In a 2018 study conducted by Ebrahimi et al., palliative radiation was found to achieve stable or decreased analgesic requirements [35]. Hypofractionated doses such as 20 Gy in 5, 30 Gy in 10, 16 Gy × 2 (weekly), 21 Gy in 3 fractions (day 1, 7, 21) may be used for palliation.

Question 4:

What is the role of SBRT in unresectable LAPC?

- SBRT can be considered for patients who have localized disease and for whom surgery and chemotherapy is contraindicated or declined. (Level III)

Evidence Summary:

SBRT in the treatment of unresectable pancreatic ductal adenocarcinoma has been shown to achieve local control however survival rates remain poor. SBRT can be considered for otherwise well, elderly patients who have localized disease only. This should only be considered in centers with expertise and plan peer review. A 2022 study conducted by Vornhulz et al found SBRT to be effective in reducing pain and improving jaundice by achieving local control [36]. A systematic review of 19 papers also found that patients who received SBRT had reductions in analgesic requirements and that some even achieved complete pain response [37]. Thus, after chemotherapy in localized unresectable pancreatic cancer without nodal disease, hypofractionated radiotherapy or SBRT with at least 30–40 Gy in 5 fractions can be considered if bowel tolerance doses are met. Total dose should be modulated depending on the anatomical location of tumor and available technical modalities.

## 6. Systemic Therapy for Hepatocellular Carcinoma 

Question 1:

What are the recommended first line systemic therapies for advanced HCC?

- Systemic therapy can be offered to patients with HCC that are not amenable to, or who have failed local therapy and have a Child Pugh A liver function. (Level 1)
- The optimal 1st line therapy should be determined by patient comorbidities, patient preference (intravenous therapy vs. oral therapy), immunotherapy contraindications, transplant status and presence of bleeding risk (untreated varices).
- The favored first line systemic therapy is Atezolizumab + Bevacizumab. Durvalumab + Tremelimumab is favored if there is a contraindication to Bevacizumab. (Level III)
- Lenvatinib or Sorafenib are favored if there is a contraindication to immunotherapy. We endorse the use of lenvatinib over sorafenib based on toxicity profile and efficacy. (Level III)

Evidence Summary:

Therapeutic choices for HCC are essentially determined by a patient’s initial functional state, comorbidities (include bleeding risks), the degree of the disease at presentation, transplant status, and the underlying liver function based on Child-Pugh score [38]. Based on IMbrave 150 trial, Atezolizumab with Bevacizumab showed superior overall survival (19.2 vs. 13.4 months) and progression-free survival (6.9 vs. 4.3 months) compared to sorafenib in patients with unresectable hepatocellular carcinoma. This is the favored first line therapy if there is no contraindication to bevacizumab or immunotherapy [39]. Interestingly, the STRIDE trial showed significant improvement in OS (30.7%) by using a single dose of Tremelimumab and every four weeks durvalumab compared to sorafenib (20.2%). As a result, this combination can be considered another option in the 1st line setting, especially if there is a contraindication to Bevacizumab [40]. Sorafenib & Lenvatinib are also widely used in first line setting. They can be used if patients have contraindication to immunotherapy, prior liver transplant, or if the patient prefers to use an oral therapeutic option rather than intravenous therapeutic [41]. Lenvatinib is recommended over sorafenib due to less toxicity and improved efficacy such as disease control rate [42]. Although Child Pugh B7 class patients were not included on these Phase III studies, our consensus was to consider systemic therapy in select patients.

Question 2:

What are the second line systemic therapies that can be used for advanced HCC?

- After 1st line tyrosine kinase inhibitor (TKI), 2nd line therapies of choice include:
  - Pembrolizumab (Level II)
  - Regorafenib (Level III)
  - Cabozantinib (Level III)
- The favored 2nd line of therapy post Atezolizumab + Bevacizumab include Lenvatinib or Sorafenib, then Regorafenib or Cabozantinib.

Evidence Summary:

Pembrolizumab as second line therapy after TKIs has a favorable risk-to-benefit ratio according to KEYNOTE-224 [43]. In HCC patients who are progressing on sorafenib therapy, regorafenib (compared with placebo) is the only systemic therapy that has demonstrated improved survival in patients who had tolerance to prior sorafenib based on the RESORCE trial [44]. The CELESTIAL trial concluded that cabozantinib extended overall survival and progression-free survival compared with placebo in patients with previously treated advanced hepatocellular cancer [45]. Based on multi-cohort study, patients on atezolizumab-bevacizumab responded effectively to second line TKI (sorafenib or Lenvatinib) with acceptable toxicity [46]. Following second line TKI, regorafenib or cabozantinib may be used in fit patient with good liver function since clinical trials have shown clinical benefit [45,47].

## 7. Role of IO in the treatment of gastric and GEJ cancers

Question 1:

What is the role of immunotherapy in the curative setting of esophageal and gastroesophageal junction carcinomas?

- In the curative setting of esophageal and gastroesophageal junction carcinomas EC/GEJ, we endorse the use of nivolumab post neoadjuvant concurrent chemotherapy and radiation and surgery for patients with residual disease based on the improved disease-free survival (DFS) and distant metastasis-free survival (DMFS) (Level 1).

Evidence Summary:

In the curative setting, patients with gastroesophageal malignancy with residual disease following neoadjuvant chemoradiotherapy (CRT) and surgical resection should receive adjuvant immunotherapy with nivolumab as per CHECKMATE-577. Patients with residual disease in this trial were randomized to receive placebo or nivolumab following CRT and definitive surgical management. Those in the nivolumab arm demonstrated a doubling in median disease-free survival compared to placebo (22.4 months versus 11.0 months, HR 0.69, *p* = 0.0003), as well as a significant decrease in distant and locoregional recurrences (DMFS 28.3 vs. 17.6 months respectively, HR = 0.74), establishing adjuvant nivolumab as a standard of care option in this population [48]. 

There are ongoing trials evaluating the addition of immunotherapy to chemotherapy in the neoadjuvant setting in patients with gastric and gastroesophageal cancers. In patients with resectable gastric/GEJ adenocarcinoma that are MSI-H, the role of immunotherapy alone is emerging and treatment with an IO agent should be considered. Discussion regarding the potential lack of benefit derived with chemotherapy for this population should be discussed in the peri-operative setting.

Question 2:

What is the role of immunotherapy in esophagogastric carcinomas in the non-curative setting?

- Nivolumab or pembrolizumab in combination with chemotherapy (fluoropyrimidine + platinum) improves the OS for patients with metastatic, unresectable esophagogastric cancers. All patients with dMMR/MSI-High tumors should be treated with immunotherapy +/− chemotherapy. (Level I)
- Combined Positive Score (CPS) for adenocarcinomas and preferentially Tumor Proportion Score (TPS) for squamous cell carcinomas predict the magnitude of benefit of immunotherapy + chemotherapy and should be considered when choosing the optimal first-line therapy. (Level I)
- Patients with adenocarcinomas and CPS score < 5 do not derive benefit from immunotherapy, and it is not recommended in this population. (Level I)

Evidence Summary:

In the non-curative setting, immunotherapy in addition to chemotherapy (fluoropyrimidine + platinum) has been shown to be superior to chemotherapy alone and should be considered for patients with metastatic gastric and GEJ cancer/esophageal adenocarcinomas without contraindications to immunotherapy. This is supported by the three-year follow-up data reported from CHECKMATE 649. In this trial, patients with untreated, unresectable esophageal/GEJ/gastric cancers, that were HER2 negative were randomised to chemotherapy alone (XELOX or FOLFOX) or chemotherapy plus the addition of PD-1 inhibitor nivolumab. They were stratified by PDL1 less than or greater than 1%. There was a clinically meaningful overall survival benefit with chemotherapy and immunotherapy (13.7 months versus 11.6 chemotherapy alone, HR = 0.79) in all randomized patients. This benefit was greater for those with CPS ≥5 (14.4 versus 11.1 respectively, HR = 0.70). Patients with MSI-H tumors were found to have the greatest OS benefit (38.7 months versus 12.3, HR = 0.34) [49]. Another trial evaluated the efficacy of pembrolizumab plus chemotherapy in treatment naive, unresectable, or metastatic esophageal/GEJ/gastric cancer population, yielding similar results. KEYNOTE 590 stratified patients by adenocarcinomas and squamous cell carcinomas and randomly assigned them to pembrolizumab plus chemotherapy or chemotherapy alone. Those in the IO + chemotherapy group demonstrated improvements in overall survival as well as progression free survival (mOS 12.4 months versus 9.8 months, HR = 0.73 and PFS 6.3 months versus 5.8 months HR = 0.64). Pre-specified subgroup analyses demonstrated benefit across all patients with CPS ≥ 10, with greatest magnitude of benefit for squamous cell carcinomas with CPS ≥ 10 (mOS 13.9 versus 8.8 months with chemotherapy alone, HR = 0.59) [50]. For squamous cell carcinomas, treatment with chemotherapy plus pembrolizumab is recommended while adenocarcinomas should be treated with chemotherapy plus nivolumab.

A recent trial evaluated the combination immunotherapy (nivolumab plus ipilimumab) versus chemotherapy or chemotherapy plus nivolumab in squamous cell carcinomas alone. Similarly, this trial demonstrated statistically significant improvements in overall survival. This benefit was greatest for those who received chemotherapy plus nivolumab, but combination immunotherapy also yielded overall survival benefit representing a new potential first line standard of care in select patients [51].

Collectively, these trials suggest that the higher the CPS score, the greater the overall survival advantage and timely CPS/TPS (for squamous cell carcinomas in particular) is therefore recommended in all metastatic or unresectable esophageal/GEJ/gastric cancers. As noted, adenocarcinomas with CPS below 5 do not seem to derive benefit so IO chemotherapy combination should not be offered, and this finding should be discussed with patients.

In patients with HER2+ metastatic or unresectable esophageal/GEJ/gastric cancers, there is a question as to the benefit of immunotherapy added to standard of care HER2 directed therapy plus chemotherapy (platinum plus fluoropyrimidine). To investigate this, patients were randomized to receive pembrolizumab or placebo in addition to chemotherapy plus trastuzumab. While results of overall survival and progression free survival are not yet mature, pembrolizumab in addition to trastuzumab and chemotherapy demonstrated and overall response rate of 74.4% versus 51.9% with chemotherapy and trastuzumab alone, and adverse events were reported as similar between arms [52]. This supports another promising future application of immunotherapy for metastatic or unresectable esophageal/GEJ/gastric cancers that are HER2+.

Question 3:

What are the important biomarkers in esophagogastric cancers?

- PDL1 testing is required for all esophagogastric cancers. (Level III)
- CPS is recommended for adenocarcinomas
- TPS and CPS are recommended for squamous cell carcinomas
- HER2 neu and MSI testing are necessary for all adenocarcinomas. (Level III)

Evidence Summary:

As per KEYNOTE-590, in patients with unresectable esophageal adenocarcinomas, median overall survival was longer with pembrolizumab in addition to chemotherapy compared to chemotherapy alone in patients with CPS ≥ 10, (HR = 0.64; 95% CI, 0.51–0.80), compared to all patients (HR = 0.73, 95% CI, 0.63–0.86) This overall survival benefit was greater for squamous cell carcinomas (HR = 0.59; 95% CI, 0.45–0.76) when compared to adenocarcinomas HR = 0.73 (95% CI, 0.55–0.99) [50]. Longer follow-up survival results of chemotherapy plus nivolumab in CHECKMATE-649 (which initially demonstrated overall survival benefit for those with CPS above and below five) presented during the 2022 ASCO Gastrointestinal Cancers Symposium, revealed the lack of overall survival advantage in patients with CPS < 10 [49]. 

CPS does correlate with efficacy to chemotherapy with immunotherapy, and it is important to note that patients with CPS < 1 unlikely to gain benefit. It is therefore recommended that CPS (or TPS) be reported for all gastroesophageal cancers as a number as cutoffs are variable (i.e., ≥1, ≥5, ≥10). Institutions must consider testing availability, funding, and tissue preservation amongst other logistics in close consultation with pathology departments. 

As per established standards of care, all adenocarcinomas should undergo testing for HER2 neu and microsatellite instability as results directly impact treatment considerations. 

## Data Availability

Not applicable.
